# Comment on: Association between intrarenal venous flow from Doppler ultrasonography and acute kidney injury in patients with sepsis in critical care: a prospective, exploratory observational study

**DOI:** 10.1186/s13054-023-04625-0

**Published:** 2023-08-29

**Authors:** Nikolaus Schreiber, Michael Kolland, Philipp Eller, Alexander R. Rosenkranz, Alexander H. Kirsch

**Affiliations:** 1 Department of Internal Medicine, Division of Nephrology, Medical University of Graz, Auenbruggerplatz 15, 8036 Graz, Austria; 2 Department of Internal Medicine, Intensive Care Unit, Medical University of Graz, Graz, Austria

Dear Editor,

With great interest we read the recent article by Fujii et al. [1], which brings forth valuable insights in the domain of volume assessment in critically ill patients. The authors conclude that distinct intrarenal venous flow (IRVF) patterns are indicative of renal venous congestion and that they do not correlate with central venous pressure (CVP) but are associated with subsequent acute kidney injury (AKI) in critically ill patients with sepsis. While recognizing the strengths inherent in this study, we strongly believe that there are several limitations that warrant a more comprehensive discussion.

Firstly, the findings diverge from the outcomes published in the study conducted by Spiegel et al., where no discernible correlation between major adverse kidney events at 30 days (MAKE30) and IRVF patterns was established [2]. The authors of the current study justify their findings based on the temporal aspect; they posit that while Spiegel et al. evaluated IRVF during an early juncture in the disease progression, Fujii et al. examined IRVF patterns subsequent to the initial phase of resuscitation, 24 h after sepsis onset. Furthermore, it is worth noting that the patient cohort in the study by Spiegel et al. exhibited a higher degree of heterogeneity and, contrary to the cohort of Fujii et al., did not exclusively include mechanically ventilated patients. This aspect merits attention since mechanical ventilation does alter venous congestion [3]. Accordingly, the cohort of Fujii et al. exposed noteworthy statistically significant differences in positive end-expiratory pressure (PEEP) levels among patients with distinct continuous and discontinuous IRVF patterns (even in this small sample). Specifically, individuals exhibiting a non-continuous IRVF pattern demonstrated elevated PEEP levels in contrast to those with a continuous pattern. Interestingly, the authors did not adjust their statistical models for this variable, neither for their primary outcome of central venous pressure (CVP) (ANCOVA adjusted for APACHE-II), nor for their secondary outcomes (generalized estimating equation with inclusion of APACHE II score and baseline KDIGO stage). Surprisingly, this aspect is not discussed, leaving open the possibility that the discontinuous IRVF might merely stem from higher PEEP values. This holds especially true considering recently published results encompassing over 25,000 patients in the MIMIC-III database, which strongly imply a robust correlation between elevated PEEP values, venous congestion, and AKI [4].

Secondly, and arguably of utmost significance, Fujii et al. omitted the consideration of fluid responsiveness at the timepoint of IRVF-assessment and nevertheless conclude that their findings deliver a potential rationale to guide post-resuscitation fluid therapy and removal in sepsis. They arbitrarily defined the initial resuscitation phase as the first 24 h hours after sepsis onset. Strong prerequisites for the right time to start fluid withdrawal have been proposed based on simple physiological principles: Beside adequate tissue perfusion, patients must not be preload responsive, because if a patient is on the steep part of the Frank–Starling curve, fluid withdrawal can lead to a decrease in cardiac output [5]. The fulfillment of these prerequisites is necessary to justify more comprehensive evaluation of venous stasis to initiate and guide fluid withdrawal. The absence of any consideration of fluid responsiveness highly damped our enthusiasm for the present study findings.

In summary, this study provides only a preliminary exploration into the complex cardio-pulmo-renal interactions it seeks to investigate, especially in the context of fluid responsiveness and venous congestion. It illustrates that assessment of fluid status remains challenging and will likely never be based on a single parameter. Ultrasonography represents an attractive tool in assessing fluid status due to its non-invasiveness as well as rapid and near universal availability in the ICU setting [1, 2, 5]. However, this study (and our raised concerns) should remind readers that most studies on ultrasound-guided volume assessment in critically ill patients are relatively small, often exploratory, and susceptible to various sources of bias.

Therefore, these findings need replication in adequately powered studies with increased sample size and thoughtful covariate selection for appropriate adjustment of statistical models to finally gain robust and generalizable results that may become routine part of clinical care.

## Data Availability

Not applicable.

## Abbreviations

AKI: Acute kidney injury
ANCOVA: Analysis of covariance
APACHE II: Acute physiology and Chronic Health Disease Classification System II
CVP: Central venous pressure
IRVF: Intrarenal venous flow
MAKE30: Major adverse kidney events at 30 days
MIMIC III: Multiparameter intelligent monitoring in intensive care
PEEP: Positive end-expiratory pressure
KDIGO: Kidney disease improving global outcomes
